# Language abilities and associated risk factors of school-aged children with cleft lip and palate

**DOI:** 10.1371/journal.pone.0299095

**Published:** 2024-04-22

**Authors:** Lim Hui Hui, Eh Yee Ling, Yazmin Ahmad Rusli, Goh Bee See, Hasherah Mohd Ibrahim

**Affiliations:** 1 Speech Sciences Program, School of Rehabilitation Sciences, Faculty of Health Sciences, Universiti Kebangsaan Malaysia (UKM), Kuala Lumpur, Malaysia; 2 Department of Otorhinolaryngology, Head & Neck Surgery, Faculty of Medicine, Hospital Canselor Tuanku Muhriz, UKM, Kuala Lumpur, Malaysia; Universiti Malaya Fakulti Perubatan: University of Malaya Faculty of Medicine, MALAYSIA

## Abstract

Previous research on children with cleft lip and palate (CLP) reported unequivocal findings with regard to language skills, with the majority suggesting persistent difficulties in early childhood. While expressive language deficits improved with age, receptive language skills were consistently lower than peers. Further study investigating the long term and persistent impact of language deficits amongst school-aged children with CLP is warranted. This was a cross-sectional study, aimed to determine the language abilities and explore the associated risk factors in Malay speaking children with CLP in Malaysia. Fifty-two children with CLP aged 7- to 12-year-old participated in this study. Language skills were assessed using the Malay Preschool Language Assessment Tool and the adapted Subway–School–age Language & Assessment Measures. Findings revealed that 14 (26.92%) school-aged children with CLP demonstrated language deficits. Children with CLP performed significantly poorer in reading comprehension (p = 0.031) and narrative (p = 0.026) skills. It was found that the age significantly influenced total receptive language score (β = 0.421, p = 0.003) and total expressive language score (β = 0.477, p = 0.000). Findings suggested that children with CLP may continue to have persistent language deficits into their school-age years. Recommendations for regular monitoring of language performance especially for those from younger age groups is warranted to help maximize school attainment.

## Introduction

Delayed vocabulary acquisition in children with cleft lip and palate (CLP) increases the risk of persistent language delay [1,2], reading problems [3], and learning difficulties [4]. A large body of evidence reported that children with CLP, as young as 2 years old, have poorer language skills compared to their typically developing peers [5–9]. However, contrasting findings were also reported by some studies that found no significant difference in language skills between children with CLP and their peers by the age of 5, indicating that these children could “catch up” to their peers [10,11]. This trend contrasted with the findings from a recent meta-analysis involving 955 young children (birth to 8 years old) from 31 studies [12]. The results suggested that children with non-syndromic cleft lip and palate (NSCLP) had significantly poorer receptive and expressive language skills than their peers without NSCLP which can persist up to the age of 8 years. It was also highlighted that the findings were influenced by methodological differences revealing that children’s age and the type of assessment utilized significantly influenced how well language was developing. Children’s expressive language caught up with peers as age increased, but the receptive language delay persisted throughout early school age. It was also noted that the disparities in language performance between children with and without CLP were more pronounced based on observational and parent reports compared to the standardized test results.

The number of studies on school-aged children with CLP is limited, with overall findings reporting that school-aged children with CLP showed a delay in at least one area of language performance. These studies reported a prevalence of language difficulties ranging from 14% [10] to 23.6% [13] on receptive and expressive language subtests. Large-scale studies of over 500 participants with CLP reported weaker academic skills, particularly reading, language and mathematics [14,15]. This is supported by a recent systematic review of academic outcomes in children with clefts revealed that reading ability is highly affected in school-aged children with language and phonological deficits, particularly in children with additional hearing loss [16]. A large study in Norway involving 184 10-year-old children with CLP revealed additional developmental disorder (e.g.: ADHD and developmental difficulties) was the only significant predictor of language and reading outcomes. Children with CLP only have a higher chance to score above normal range than children with CLP associated with additional developmental disorders. However, the percentage of children with CLP only that scored below the normal range in the tests was high: total language screening (44.7%), word chain (36.4%), and reading comprehension (33.3%) [13]. The absence of a control group and under-reported hearing threshold should be examined to explain the high prevalence of language and reading difficulties. Boyce et al. [10] and Morgan et al. [17] reported 14% to 20% of school-aged children with CLP scored ≥ 1.25 SD below the mean in one or more language index on the CELF-4 language assessment, indicating language impairments.

Risk factors for the delay in these children’s language skills have primarily been associated with hearing status, cleft type, cognitive performance, and age of palatal repair. However, there is no consensus over the relative contributions of these factors to language delay or impairment. Abnormal middle ear status was highly prevalent in children and young adults with CLP than typically developing peers [18,19]. Specifically in Malaysia, 25% to 30% of Malaysian children with CLP were reported to present with hearing loss in two separate studies [20,21]. Although abnormal middle ear status decreased significantly with age, the hearing threshold in the higher frequencies did not improve [22], leading to a challenge in educational attainment such as reading and speaking [23]. However, there were studies reporting contrasting findings that hearing status did not predict language and reading scores [7,12,13,23–25]. Cognitive development and non-verbal IQ proved to be robust predictors of later language functioning [10,16,26].

Studies on Malaysian children with CLP have been focused on examining their speech profiles (i.e.: speech intelligibility, nasality, phonological process), particularly on Malay-speaking children in urban cities such as Kuala Lumpur and Kelantan [27,28]. There are no studies exploring the language performance of children with CLP in school age. To address these research gaps, the present study aims to identify the prevalence of language disorders and their risk factors in Malay speaking children with CLP. Specifically, this study aims to (1) identify the language abilities in school-aged children with CLP, (2) compare the differences in language abilities between school-aged children with CLP and their age-matched peers, (3) explore the association between socio-demographic factors and children’s language abilities.

## Materials and methods

### Participants and study location

This was a cross-sectional study. Participants with CLP were recruited from Cleft Lip and Palate Association Malaysia (CLAPAM) and through a teaching hospital, Hospital Canselor Tuanku Muhriz Universiti Kebangsaan Malaysia (HCTM). Inclusion criteria included: (1) age 7- to 12- years old; (2) attending public primary school; (3) native speakers of Malay. Those diagnosed with syndromic CLP or any form of complex medical conditions as reported by parents were excluded. Demographic and developmental information such as age, cleft type, speech therapy attendance, maternal education, and socio-economic status were obtained via parental reports and questionnaires. In total, fifty-three children with CLP were recruited. They completed pure tone audiometry (PTA) to assess hearing level. In addition, twenty-five typically developing children were recruited as the control group. The inclusion criteria included (1) children between the age of 7- to 12- years old, (2) attending public primary school (3) native speakers of the Malay language, and (4) having no history of language problems and craniofacial anomalies as reported by their parents. The control group was selected on a voluntary basis using a convenience sampling method from students attending local public primary schools. The study was approved by the Human Research Ethics Committee of Universiti Kebangsaan Malaysia (JEP2021-875) and conducted in accordance with the Helsinki Declaration (as revised in 2013). Parental consents were obtained for ethical purposes. The information of participants was anonymised during the data collection and reported collectively with no reference to an individual.

### Sample size calculation

The sample size was calculated using G*Power version 3.1.9.4. An effect size of 0.35 and a statistical power (1-β error probability) of 0.90 were used in a linear multiple regression statistical test. The estimated sample size for this study was 53 subjects.

### Materials and general procedure

Children were seen at Universiti Kebangsaan Malaysia’s Audiology and Speech Clinic, while those who could not attend physically were offered online sessions. Their speech intelligibility was rated based on the 5-point rating scale in Cleft Audit Protocol for Speech (CAPS) [29]. The scoring and definitions of the speech intelligibility scale are as follows: 0 = normal, 1 = different from other children’s speech, but not enough to cause a comment, 2 = different enough to provoke comment, but possible to understand most speech, 3 = only just intelligible to strangers, 4 = impossible to understand.

Children also completed five language tasks which included receptive and expressive language skills. Two were taken from the Malay Preschool Language Assessment Tool (MPLAT) [30], namely: (1) grammatical comprehension (receptive skill) which required children to identify pictures which matched the tested sentences, (2) sentence repetition (expressive skill) where children were asked to repeat the sentence that they heard verbatim. MPLAT is a standardized normed referenced language assessment tool for Malay preschoolers aged 4;0–6;11 years old. This test was chosen because there was no standardized norm-referenced language assessment for Malay school-aged children [30,31] Thus, 25 typically developing school-aged children were recruited as the control group to compare their language scores. Two tests from Razak et al. [32] were used to assess reading comprehension and listening comprehension. Additionally, children also completed a narrative assessment using the adapted The Subway–School–Age Language and Assessment Measures (SLAM) [33]. In this task, children were asked to narrate, reflect, and make inferences based on a picture of the subway.

#### Language outcome

Language impairment was defined as performance 2 SD below the mean of the control group. Cut-off scores were therefore as follows: 13.82 (grammatical comprehension); 5.92 (reading comprehension); 0.77 (listening comprehension); 3.42 (sentence repetition); and 4.55 (narrative test (SLAM)). A child from the CLP group would fail a language task if they scored below the cut-off score. Children’s language performance was classified as age-appropriate if they scored above the cut-offs on all the language measures administered. They were classified as being at risk of having language difficulties if one of their language scores fell below the cut-offs and classified as having language difficulties if they failed on two or more of the language measures.

### Variables

The primary outcome measure was the total language score (receptive and expressive language). Independent variables or predictors were age, type of cleft, presence of hearing loss, maternal education, and speech intelligibility. SES and maternal education status were determined through a self-reported questionnaire where parents selected their highest education level and monthly household income based on choices provided.

### Data analysis

Data analysis was carried out using IBM SPSS (Statistical Package for the Social Sciences) software version 26.0. Descriptive statistical analyses were used to describe the demographic information including age, type of cleft, presence of hearing loss, maternal education and speech intelligibility. An independent T-test was used to determine the difference between language scores of the cleft and control groups. Multiple linear regression was used to determine the association between language scores and age, cleft type, hearing loss, maternal education or speech intelligibility. The data are available at https://figshare.com/s/f9a142d1f114c5115c18.

## Results

### Demographic data

Table 1 shows the demographic background of 52 school-aged children with CLP who participated in this study. Fifty-three participants were recruited but one participant withdrew from the study due to inability to complete the tests, leaving a total of 52 participants. The mean age of the total sample was 9.19 (SD = 1.61) years old. Majority of the participants, (86.5%) presented with CLP, however only a third of them (36.5%) had hearing issues, with conductive (26.9%) and bilateral hearing loss (25.0%) making up the most common types of hearing loss. Most of the participants had either speech intelligibility that was normal (38.5%), or different from other children but was not enough to cause comment (32.7%). The majority of the participants were also from middle to low socio-economic status and lower maternal education. For the control age-matched group, 25 children with mean age 9.65 (SD = 1.62) years old were recruited. There were 11 males and 14 females.

**Table 1:** Demographic information of school-age children with CLP.

Characteristic	Subcategory	N (%)
Age in years	7	16 (30.8%)
	8	10 (19.2%)
	9	9 (17.3%)
	10	6 (11.5%)
	11	8 (15.4%)
	12	3 (5.8%)
Gender	Male	35 (67.3%)
	Female	17 (32.7%)
Type of cleft	CL	5 (9.6%)
	CP	2 (3.8%)
	CLP	45 (86.5%)
Presence of hearing loss	Absent	33 (63.5%)
	Present	19 (36.5%)
Type of hearing loss	No HL	33 (63.5%)
	Conductive HL	14 (26.9%)
	Sensorineural HL	1 (1.9%)
	Mixed HL	4 (7.7%)
Laterality of hearing loss	No HL	33 (63.5%)
	Unilateral HL	6 (11.5%)
	Bilateral HL	13 (25.0%)
Maternal Education	SPM	14 (26.9%)
	STPM	19 (36.5%)
	Degree	8 (15.4%)
	Masters	10 (19.2%)
	PhD	1 (1.9%)
Socio-economic status	Low (< rm 4850)	19 (36.5%)
	Middle (RM 4851—RM 10970)	25 (48.1%)
	High (> RM 10971)	8 (15.4%)
Speech Intelligibility	Normal	20 (38.5%)
	Different from other children, but not enough to cause comment	17 (32.7%)
	Different enough to provoke comment	10 (19.2%)
	Only just intelligible to strangers	4 (7.7%)
	Impossible to understand	1 (1.9%)
Receiving speech therapy at the time of assessment	Yes No	13 (25%) 39 (75%)

CL, cleft lip; CLP, cleft lip and palate; CP, cleft palate; HL, hearing loss; SPM, Sijil Pelajaran Malaysia (Malaysian Certificate of Education examination); STPM, Sijil Tinggi Persekolahan Malaysia (Malaysian Higher School Certificate examination).

### Language assessment

Table 2 shows the descriptive statistics of all language tasks administered, with the measures divided into receptive and expressive domains. Significant mean differences with medium effect size were found in two of the five language measures: reading comprehension (p = 0.031), d = 0.588 and narrative (SLAM) (p = 0.026), d = 0.578.

**Table 2:** Mean score differences between cleft group and control group in the language performance.

Language component	Sub–test	Cleft (n = 52) mean (SD)	Control group (n = 25) mean (SD)	p < 0.05	Mean difference	(95% CI)	Cohen’s d
Receptive language	Grammatical comprehension	17.10 (1.785)	17.64 (1.912)	0.225	-0.544	(-1.43, 0.342)	0.292
	Reading comprehension	7.29 (2.460)	8.44 (1.261)	0.031*	-1.152	(-2.194, -0.109)	0.588
	Listening comprehension	3.00 (1.138)	3.24 (1.234)	0.402	-0.240	(-0.807, 0.327)	0.202
Expressive language	Sentence repetition	11.04 (4.785)	13.24 (4.91)	0.065	-0.202	(-4.541, 0.138)	0.454
	Narrative (SLAM)	6.12 (1.567)	6.92 (1.187)	0.026*	-0.805	(-1.511, -0.098)	0.578

*Significance level is at p < 0.05.

### Language outcome

Table 3 shows the number of subjects being at risk of having language difficulty. Fourteen (27%) of school-aged children with CLP in this study were reported to have poor language skills compared to the control group. Specifically, eight (15%) were at risk of having language difficulties in at least one subtest (failed one subtest), and six (12%) were identified as having difficulties in both receptive and expressive subtests (failed two or more subtests in receptive and expressive subtests).

**Table 3:** Demographic details and language subtests scores of subjects found being at risk of having language difficulty and having language difficulty.

Age	Cleft type	Presence of Hearing Loss	Receptive Language (Comprehension)			Expressive Language		**Language Abilities
			Grammar *(13.82)	Reading *(5.92)	Listening *(0.77)	Sentence Repetition *(3.42)	Narration *(4.55)	
7	CLP	No	15	5	2	12	6	At risk
7	CLP	No	18	1	2	9	5	At risk
8	CLP	No	16	9	2	5	4	At risk
8	CLP	Yes	18	8	3	13	3	At risk
9	CLP	Yes	13	9	4	9	6	At risk
10	CL	No	19	5	5	17	8	At risk
10	CLP	Yes	15	8	2	15	4	At risk
11	CLP	Yes	18	8	3	6	4	At risk
7	CLP	Yes	15	5	0	3	3	LD
7	CLP	Yes	17	1	2	2	6	LD
7	CLP	No	17	1	1	3	6	LD
7	CLP	Yes	11	0	1	0	4	LD
8	CLP	Yes	17	4	2	16	2	LD
8	CLP	No	16	3	1	7	3	LD

Note: CL, cleft lip; CLP, cleft lip and palate; CP, cleft palate; LD, language difficulties

*cut-off score for each language subtest

** At risk of having language difficulties = One language subtest score fell below the cut-offs; Having language difficulties = Two or more language subtests scores fell below the cut-offs

### Risk factors of language performance

Separate multiple linear regressions were performed to determine the total amount of variance that age, type of cleft, presence of hearing loss, maternal education and speech intelligibility contributed to children’s receptive and expressive language scores. Tables 4 and 5 show the unstandardised (B) and standardised (β) regression coefficients for each predictor in a regression model predicting total receptive and expressive language score. For children’s receptive language scores, the predictors jointly accounted for 22.1% of the total variance (R2 = 0.221, adjusted R2 = 0.136), F (5, 46) = 2.610, p = 0.037, f = 0.284. Age accounted for 4.21% of unique variance, while contributions from the remaining predictors; type of cleft (0.11%), presence of hearing loss (-1.82%), maternal education (0.86%) and speech intelligibility (0.86%) were statistically nonsignificant. Similarly, for children’s expressive language scores, the predictors jointly accounted for 30.8% of the total variance (R2 = 0.308, adjusted R2 = 0.233), F (5, 46) = 4.103, p = 0.004, f = 0.445. Again, only age accounted significantly for 4.77% of unique variance.

**Table 4:** Unstandardised (B) and standardised (β) regression coefficients for each predictor in a regressiom model predictin total receptive language score.

Variables	B [95% CI]	β	p < 0.05
Age	1.054 [0.387, 1.720]	0.421	0.003*
Type of cleft	0.071 [-1.585, 2.000]	0.011	0.941
Presence of hearing loss	-1.531 [-3.890, 0.829]	-0.182	0.198
Maternal Education	0.309 [-0.669, 1.288]	0.086	0.528
Speech Intelligibility	0.339 [-0.779, 1.457]	0.086	0.545

Dependent Variable: Total Receptive Language Score

Note. N = 52. CI = confidence interval

*Significance level is at p<0.05.

**Table 5:** Unstandardised (B) and standardised (β) regression coefficients for each predictor in a regressiom model predicting total expressive language score.

Variables	B [95% CI]	β	p < 0.05
Age	1.635 [0.775, 2.495]	0.477	0.000*
Type of cleft	-1.906 [-4.395, 0.584]	-0.208	0.130
Presence of hearing loss	-2.179 [-5.224, 0.867]	-0.189	0.157
Maternal Education	-0.199 [-1.462, 1.065]	-0.040	0.753
Speech Intelligibility	0.23 [-1.213, 1.673]	0.043	0.750

Dependent Variable: Total Expressive Language Score

Note. N = 52. CI = confidence interval

*Significance level is at p<0.05.

## Discussion

This is the first study to investigate the language proficiency and the associated risk factors in Malay speaking school-aged children with CLP.

The results indicated that children with CLP in this study showed significant differences in reading comprehension (p = 0.031) and narrative (p = 0.026) compared to the control group. While children’s mean language performances were similar on the remaining grammatical understanding, listening comprehension and sentence repetition tasks, the CLP group demonstrated lower mean scores comparatively across all language tests. The majority of the school-aged children with CLP in this sample had normal language skills, and the remaining 14 children (27%) have language impairment. This number is higher than the studies done by Boyce et al. [10] and Morgan et al. [17] where 14.1% and 20% of their children with CLP have a mild language delay in at least one domain of language functioning, based on the Clinical Evaluation of Language Fundamentals, Fourth Edition (CELF-4). This may be due to the recruitment of a younger cohort of children (mean age = 5.91 years old) which allowed for greater influences from middle-ear abnormalities and speech difficulties, both of which are frequently found to be more common in younger age groups with CLP [18,34]. Since the current study uses criterion referenced tests, there may also be limitations when comparing the results with existing studies due to the different nature of language assessment tools used.

In terms of literacy skills, the findings of this study are comparable to the findings of the systematic review conducted by Gallagher & Collett [16] who reported that children with orofacial clefts have difficulties particularly in reading and language dependent subjects. The systematic review specifically showed that children in early school years showed below average competencies than peers in reading comprehension.

For the expressive language subtests, children with CLP in this study performed poorer than the control group. Significant differences were noted for narrative tasks. Although previous studies showed no difficulties with narrative tasks [10,13], within group differences especially in children with CLP with other comorbidities such as hearing issues must be interpreted with caution. Subjects in our study included all children with hearing and speech issues which may have contributed to the poorer scores.

Besides, children in the current study showed typical grammatical understanding, different from findings from Boyce et al. [10], reported the lowest scoring subtest for the cleft group was sentence structure, the understanding of grammatical functions in spoken sentences of varying length and complexity. Morgan et al. [17] however reported total language score instead of subtest score and found that the children did better in receptive language compared to expressive language tasks. Interestingly, children with CLP as a group in our study performed at par with the typically developing control group’s performance on listening comprehension and sentence repetition tasks. These results were comparable to previous studies where children did well in sentence repetition [10,13].

### Effects of age on language outcomes

This study revealed that only age significantly influenced total receptive language score (β = 0.421, p = 0.003) and total expressive language score (β = 0.477, p = 0.000) in school-aged children with CLP. Other socio-demographic factors (i.e., cleft type, presence of hearing loss, maternal education level and speech intelligibility) did not significantly predict language or literacy performance in school-aged children with CLP.

A systematic review by Lancaster and colleagues [35] showed that age did not moderate reading development in young and school-aged children with CLP although this population performed persistently poorer in reading tasks than peers. In a separate study of meta-analysis, Lancaster [12] revealed age was a significant moderator of expressive language in young children with CLP, but not receptive language. This indicates that young children with CLP ’catch up’ on their expressive language as their age increases. However, the findings of the current study suggested age was a significant moderator influencing both total receptive (reading and listening comprehension, grammatical understanding) and expressive language (narration, sentence repetition) scores of school-aged children with CLP.

We found that a significantly higher number (79%) of children from the younger age group (7 to 9 years) were detected to be either at risk or having language delay than (21%) of children from the age group (11 to 12 years). One of the reasons 7- and 8-year-old children in this study struggled with the language tests was that many did not attend school for 2 years during the Covid-19 outbreak in 2020. Classes were conducted online for two years and children have difficulty in transition from kindergarten to primary school in Malaysia. Since parents were working from home at the time of lockdown, home literacy practice (e.g., reading together more than 15 minutes in a week, talking about what happened in the story etc.) with children with CLP will be a contributing factor in supporting literacy development and preventing reading difficulty in later school age [36].

Three (21%) children aged 10–11 years old were identified to be at risk of having language difficulties. Two of the children failed in the hearing test, have low socio-economic status and low maternal education level. However, a large study in Norway revealed that (33.3–44.7%) of the 10-year-old Norwegian children with CLP only scored below the normal range in language and reading tasks [13]. This study reported a higher number of children with language impairments, probably also due to the large sample size. Moreover, out of 14 children who were at risk and having language difficulties, only one is still attending speech therapy, while 13 have never received speech therapy, discontinued or have been discharged from respective speech therapy teams.

The regression model in the study accounted for a small amount of variance in the total receptive language score and in the total expressive language score. Therefore, the significant predictor, age of the children does not explain the overall language performance in school-aged children with CLP alone.

### Effects of hearing on language outcomes

Our analysis showed no significant relationship between the presence of hearing loss and receptive and expressive language scores. Mixed findings were reported on the role of hearing in children with CLP. Our results tallied with a large number of studies which found no relationship between hearing and language or reading development in preschoolers and school-aged children with CLP [7,13,23–25]. A small number of studies reported hearing level significantly predicted the development of vocabulary skills in children aged 18 to 24 months old [37]. Systematic review conducted by Hall et al. [23] also revealed hearing status might influence reading skills in school-aged children with CLP. Due to different methodology sampling, some studies reported incomplete hearing data in their retrospective study [25], some do not include children with hearing loss particularly those with sensorineural hearing loss or moderate conductive hearing loss, thus putting a barrier to discuss the role of hearing status in predicting the language skill in children [5,10]. Thus, the relationship between hearing status and language development in children with CLP remained inconclusive.

### Effects of cleft types on language outcomes

The impact of cleft type on language development has yielded conflicting results. There were studies which reported no relationship between cleft types and language outcomes [10,25]. Children with CP (cleft palate) have a higher chance to be delayed in major literacy and language tests in recent population based studies [14,15]. Some studies did not examine the language skills in children with CL (cleft lip) as the assumption was they have normal speech and language skills due to the intact palate [9–11,13,17]. However, children with CL had similar likelihood for reaching minimum standards in literacy and language tests with children with CLP and CP [14,15]. Our studies did not find any relationship between cleft types and language outcomes, probably due to the uneven distribution of cleft type in our participants, with the majority of them having CLP (n = 45), and a small sample of children having CP (n = 2) and CL (n = 5).

### Effects of speech intelligibility on language outcomes

No significant evidence was found in association of the overall language performance in receptive and expressive language with speech intelligibility in our study. This is supported by a recent study which had reported the presence of articulation errors in preschoolers with CLP did not appear to have an effect on language impairment [7]. In contrast, Pamplona and Ysunza [38] and Pamplona et al. [39] found that children with CLP with compensatory articulation (CA) have deficits in language. Lancaster et al. [35] postulated that speech production errors might affect reading development in children with CLP, but further research was needed to examine the effects of phonological awareness on reading. Overall, limited study was done on association of language skills, speech and reading in children with CLP population [26].

## Strengths and limitations

This is the first study that utilized a larger sample than previous local studies on CLP, to examine the receptive and expressive language skills of Malay speaking school-aged children with CLP. While previous studies have focused on expressive language difficulties in children with CLP, the findings of this study support that children with CLP may exhibit difficulties in both receptive and expressive language skills. Nevertheless, it is imperative to delineate the limitations within the scope of this study. The absence of standardized assessments in the Malay language specifically tailored for school-age children prompted the utilization of a criterion-referenced approach to gauge the language proficiency of children with CLP, contrasting their performance against a control group. It is essential to highlight that the control group, although age-matched, was recruited voluntarily, potentially introducing bias in result interpretation due to the smaller sample size and uncontrolled confounding variables such as socioeconomic status and gender. Secondly, children from CLP and control groups were tested after the pandemic period which may have affected the language scores in younger age groups as schools were fully online for almost 2 years in Malaysia. Language tests were carried out through physical and online sessions based on the participants’ availability. Language performance and the presentation of the language stimuli (i.e., audio in listening test) may influence the language test results. In this study, the inter-rater reliability was not measured although the language test was administered by trained speech-language therapists from Universiti Kebangsaan Malaysia. Caution is warranted against overinterpreting results, given the potential impact on language outcomes. Even though all cleft types were included in this study, the type of cleft in the participants was not balanced. Subgroups of children with CPO (n = 2) and CLO (n = 5) was too small compared to CLP (n = 45) to allow a strong estimate of their risk of having language delay. In this study, speech intelligibility was measured using a descriptive rating scale and it was not corroborated by other speech measures such as nasality and articulation which is called for [40]. This may under-represent the speech abilities of the children with CLP. There remains a subset of children with CLP who experience persistent language difficulties for whom contributing factors have not been identified. Further research should address these limitations for a more comprehensive understanding of the contributing factors, particularly within the context of school-age language development.

## Conclusion

The findings of this study add to the current evidence supporting persistent language deficits in school-aged children with CLP, particularly in reading comprehension and narrative language skills. Thus, we suggest healthcare professionals especially speech language therapists include language assessment and intervention in the management of school-aged children with CLP. As language and literacy difficulties are prevalent in younger children with CLP, regular monitoring of language performance is warranted in preschool years, to scaffold children’s early literacy and language skills. Although language skills are anticipated to improve with age, further research is needed to identify the factors that moderate the language and literacy development in children with CLP (e.g., home literacy practice, language skills at 4 years of age, access to speech therapy, and cognitive profiles).

## Supporting information

S1 Checklist STROBE statement—checklist of items that should be included in reports of observational studies. (DOCX)

S2 Checklist *PLOS ONE* clinical studies checklist. (DOCX)

S1 Data (XLSX)

S1 Fig The overview of language domains tested in this study. (TIF)

## References

[1] Morris H, Ozanne A. Phonetic, phonological, and language skills of children with a cleft palate. Cleft Palate Craniofac J. 2003;40(5):460–70. doi: 10.1597/1545-1569_2003_040_0460_ppalso_2.0.co_2 12943443

[2] Priester GH, Goorhuis-Brouwer SM. Speech and language development in toddlers with and without cleft palate. Int J Pediatr Otorhinolaryngol. 2008;72(6):801–6. doi: 10.1016/j.ijporl.2008.02.004 18384888

[3] Lancaster HS, Lien KM, Haas J, Ellis P, Scherer NJ. Reading development in children with nonsyndromic cleft palate with or without cleft lip: meta-analysis and systematic review. Cleft Palate Craniofac J. 2022;59(9):1155–1166. doi: 10.1177/10556656211039871 34516236

[4] Bell JC, Raynes-Greenow C, Turner R, Bower C, Dodson A, Nicholls W, et al. School performance for children with cleft lip and palate: a population-based study. Child Care Health Dev. 2017;43(2):222–231. doi: 10.1111/cch.12388 27502161

[5] Anaraki ZG, Faham M, Derakhshandeh F, Hosseinabad HH, Haresabadi F. Language parameters of 4- to 7-year-old Persian-speaking children with cleft lip and palate. Folia Phoniatrica et Logopaedica. 2017;68(3):119–23. 10.1159/000450639 27820942

[6] Chapman KL. The relationship between early reading skills and speech and language performance in young children with cleft lip and palate. Cleft Palate Craniofac J. 2011;48(3):301–11. doi: 10.1597/08-213 20815721

[7] Young SE, Purcell AA, Ballard KJ. Expressive language skills in Chinese Singaporean preschoolers with nonsyndromic cleft lip and/or palate. Int J Pediatr Otorhinolaryngol. 2010;74(5):456–64. doi: 10.1016/j.ijporl.2010.01.014 20202695

[8] Chapman KL, Hardin-Jones M, Halter KA. The relationship between early speech and later speech and language performance for children with cleft lip and palate. Clin Linguist Phon. 2003;17(3):173–97. doi: 10.1080/0269920021000047864 12858838

[9] Eshghi M, Adatorwovor R, Preisser JS, Crais ER, Zajac DJ. Lexicogrammatical skills in 2-year-old children with and without repaired cleft palate. Clin Linguist Phon. 2022;36(6):528–46. doi: 10.1080/02699206.2021.1941263 34263689 PMC8760352

[10] Boyce JO, Kilpatrick N, Reilly S, Da Costa A, Morgan AT. Receptive and expressive language characteristics of school-aged children with non-syndromic cleft lip and/or palate. Int J Lang Commun Disord. 2018;53(5):959–68. doi: 10.1111/1460-6984.12406 29968398

[11] Collett BR, Leroux B, Speltz ML. Language and early reading among children with orofacial clefts. Cleft Palate Craniofac J. 2010;47(3):284–92. doi: 10.1597/08-172.1 20426677 PMC3397667

[12] Lancaster HS, Lien KM, Chow JC, Frey JR, Scherer NJ, Kaiser AP. Early speech and language development in children with nonsyndromic cleft lip and/or palate: A meta-Analysis. J Speech Lang Hear Res. 2019 13;63(1):14–31. doi: 10.1044/2019_JSLHR-19-00162 31841365 PMC7213476

[13] Feragen KB, Aukner R, Særvold TK, Hide Ø. Speech, language, and reading skills in 10-year-old children with palatal clefts: The impact of additional conditions. J Commun Disord. 2017;66:1–12. doi: 10.1016/j.jcomdis.2017.03.001 28292606

[14] Wehby GL, Collet B, Barron S, Romitti PA, Ansley TN, Speltz M. Academic achievement of children and adolescents with oral clefts. Pediatrics. 2014;133(5):785–92. doi: 10.1542/peds.2013-3072 24753523 PMC4006437

[15] Bell JC, Raynes-Greenow C, Turner R, Bower C, Dodson A, Nicholls W, et al. School performance for children with cleft lip and palate: a population-based study. Child Care Health Dev. 2017 Mar 1;43(2):222–31. doi: 10.1111/cch.12388 27502161

[16] Gallagher ER, Collett BR. Neurodevelopmental and academic outcomes in children with orofacial clefts: a systematic review. Pediatrics. 2019;144(1):1–10. doi: 10.1542/peds.2018-4027 31189616

[17] Morgan AR, Bellucci CC, Coppersmith J, Linde SB, Curtis A, Albert M, et al. Language development in children with cleft palate with or without cleft lip adopted from non–english-speaking countries. Am J Speech Lang Pathol. 2017;26(2):342–54. doi: 10.1044/2016_AJSLP-16-0030 28329403

[18] Flynn T, Möller C, Jönsson R, Lohmander A. The high prevalence of otitis media with effusion in children with cleft lip and palate as compared to children without clefts. Int J Pediatr Otorhinolaryngol. 2009;73(10):1441–6. doi: 10.1016/j.ijporl.2009.07.015 19709760

[19] Sheahan P, Miller I, Sheahan JN, Earley MJ, Blayney AW. Incidence and outcome of middle ear disease in cleft lip and/or cleft palate. Int J Pediatr Otorhinolaryngol. 2003;67(7):785–93. doi: 10.1016/s0165-5876(03)00098-3 12791455

[20] Cheong JP, Soo SS, Manuel AM. Factors contributing to hearing impairment in patients with cleft lip/palate in Malaysia: a prospective study of 346 ears. Int J Pediatr Otorhinolaryngol. 2016 Sep 1;88:94–7. doi: 10.1016/j.ijporl.2016.06.045 27497393

[21] Goh BS, Tang CL, Hashim ND, Annamalay T, Abd Rahman FN. Hearing status and behavioural patterns among school aged children with cleft lip and/or palate. Int J Pediatr Otorhinolaryngol. 2019 Mar 1;118:1–5. doi: 10.1016/j.ijporl.2018.12.010 30578988

[22] Flynn T, Lohmander A, Moller C, Magnusson L. A longitudinal study of hearing and middle ear status in adolescents with cleft lip and palate. Laryngoscope. 2013 Jun;123(6):1374–80. doi: 10.1002/lary.23839 23208794

[23] Hall A, Wills AK, Mahmoud O, Sell D, Waylen A, Grewal S, et al. Centre-level variation in outcomes and treatment for otitis media with effusion and hearing loss and the association of hearing loss with developmental outcomes at ages 5 and 7 years in children with non-syndromic unilateral cleft lip and palate: the Cleft Care UK study. Part 2. Orthod Craniofac Res. 2017;20 Suppl 2:8–18. 10.1111/ocr.12184 28661080

[24] Waknis A.; Karulkar R.; Limaye M.; Vanaja CS. Language development of children with cleft lip and palate. Int. J. Recent Res. Appl. Stud. 2017;4(3):2.

[25] Thanawirattananit P, Prathanee B. Relationship of language development and hearing status in children with cleft lip and palate. J Med Assoc Thai. 2013;96 Suppl 4:S49–54. . 24386742

[26] Hardin-Jones M, Chapman KL. Cognitive and language issues associated with cleft lip and palate. Semin Speech Lang. 2011;32(2):127–40. doi: 10.1055/s-0031-1277715 21948639

[27] Mohd Ibrahim H, Mohamed Yusoff FH, Ahmad K, Van Dort S. An exploratory study on speech and hearing outcomes in children with cleft lip and palate. Med J Malaysia. 2015;70(6):321–5. 26988203

[28] Normastura AR, Mohd Khairi MD, Azizah Y, Nizam A, Samsuddin AR, Naing L. Speech disorders in operated cleft lip and palate children in Northeast Malaysia. Med J Malaysia. 2008;63(1):21–5. 18935726

[29] Sell D, John A, Harding-Bell A, Sweeney T, Hegarty F, Freeman J. Cleft audit protocol for speech (CAPS-A): a comprehensive training package for speech analysis. Int J Lang Commun Disord. 2009;44(4):529–48. doi: 10.1080/13682820802196815 18821108

[30] Rogayah A.R., Neelagandan A.I., Yusuf N.M., Lim H.W., Ahmad K., & Madison C.L. The validation of the Malay Preschool Language Assessment Tool (MPLAT): the screening and diagnostic versions. Malays. J. Public Health Med. 2018;(1 Special Volume):191–115.

[31] Lim HW, Lee ST. Assessing children’ s native language in Mandarin using the adapted New Reynell Developmental Language Scales-Mandarin. GEMA Online J. Lang. Stud. 2017;17(2):123–45.

[32] Razak R A., Xing Lin L, Azmarul A. Aziz M. Oral language skills and literacy skills of Malay children with dyslexia. Learning Disabilities—Neurobiology, Assessment, Clinical Features and Treatments. IntechOpen; 2022; 1–21. Available from: 10.5772/intechopen.99787

[33] Crowley C. Leadersproject org. 2014. School-age Language Assessment Measures: The Subway. Available from: https://www.leadersproject.org/2015/03/18/slam-subway-picture-school-aged-language-assessment-measure/

[34] Nyberg J, Peterson P, Lohmander A. Speech outcomes at age 5 and 10 years in unilateral cleft lip and palate after one-stage palatal repair with minimal incision technique—A longitudinal perspective. Int J Pediatr Otorhinolaryngol. 2014;78(10):1662–70. doi: 10.1016/j.ijporl.2014.07.016 25112165

[35] Lancaster HS, Lien KM, Haas J, Ellis P, Scherer NJ. Reading development in children with nonsyndromic cleft palate with or without cleft lip: Meta-analysis and systematic review. Cleft Palate Craniofac J. 2022;59(9):1155–66. doi: 10.1177/10556656211039871 34516236

[36] Porod TK, Gorman BK. Home and Clinical Literacy practices for children with cleft lip and palate. Cleft Palate Craniofac J. 2020;57(10):1216–29. doi: 10.1177/1055665620924938 32400178

[37] Eshghi M, Adatorwovor R, Preisser JS, Crais ER, Zajac DJ. Vocabulary growth from 18 to 24 months of age in children with and without repaired cleft palate. J Speech Lang Hear Res. 2019;62(9):3413–3430. doi: 10.1044/2019_JSLHR-L-18-0207 31437085 PMC6808344

[38] Pamplona MDC, Ysunza PA. Deliberate Practice: Preliminary results of a useful strategy for correcting articulation in children with cleft palate. J Craniofac Surg. 2018;29(6):1490–1494. doi: 10.1097/SCS.0000000000004707 29916979

[39] Pamplona MC, Ysunza A, González M, Ramírez E, Patiño C. Linguistic development in cleft palate patients with and without compensatory articulation disorder. Int J Pediatr Otorhinolaryngol. 2000;54(2–3):81–91. doi: 10.1016/s0165-5876(00)00332-3 10967376

[40] Sell D. Issues in perceptual speech analysis in cleft palate and related disorders: A review. Int J Lang Commun Disord. 2005;40(2):103–21. doi: 10.1080/13682820400016522 16101269

