# Supplementary material for: Language abilities and associated risk factors of school-aged children with cleft lip and palate
Source: PLoS One. 2024 Apr 22;19(4):e0299095. doi: 10.1371/journal.pone.0299095 (PMC11034652; doi:10.1371/journal.pone.0299095)
Supplement: S1 Checklist — (DOCX) [file pone.0299095.s001.docx]

STROBE Statement—checklist of items that should be included in reports of observational studies

(Pages were numbered according to the original copy of manuscript submitted)

|  | Item No. | Recommendation | Page  No. | Relevant text from manuscript |  |
| --- | --- | --- | --- | --- | --- |
| **Title and abstract** | 1 | (*a*) Indicate the study’s design with a commonly used term in the title or the abstract | 1-2 | This is a cross-sectional study, aimed to determine the language  abilities and explore the associated risk factors in Malay speaking children  with CLP in Malaysia. |  |
|  |  | (*b*) Provide in the abstract an informative and balanced summary of what was done and what was found | 1-3 | Refer manuscript |  |
| Introduction | | | |  |  |
| Background/rationale | 2 | Explain the scientific background and rationale for the investigation being reported | 3-6 | -The number of studies in school-aged children with CLP are limited with overall findings reporting that school-aged children with CLP showed delay in at least 1 area of the language performance.  -The relative contributions of risk factors in language delay or impairment are not in consensus.  -Studies on Malaysian children with CLP have been focused on examining their speech profiles, there are no studies exploring language performance of children with CLP in school age. |  |
| Objectives | 3 | State specific objectives, including any prespecified hypotheses | 7 | (1) identify the language abilities in  school-aged children with CLP (2) compare the differences in language  abilities between school-aged children with CLP and their age-matched  peers  (3) explore the association between socio-demographic factors and  children’s language abilities. |  |
| Methods | | | |  |  |
| Study design | 4 | Present key elements of study design early in the paper | 7 | This is a cross-sectional study. |  |
| Setting | 5 | Describe the setting, locations, and relevant dates, including periods of recruitment, exposure, follow-up, and data collection | 7-8 | -Klinik Audiologi Sains Pertuturan UKM/ Online via Zoom meeting.  -Recruitment of participants started from 21 March 2022 to 30 December 2022.  -No follow up. |  |
| Participants | 6 | (*a*) *Cohort study*—Give the eligibility criteria, and the sources and methods of selection of participants. Describe methods of follow-up  *Case-control study*—Give the eligibility criteria, and the sources and methods of case ascertainment and control selection. Give the rationale for the choice of cases and controls  *Cross-sectional study*—Give the eligibility criteria, and the sources and methods of selection of participants | 7-8 | **Inclusion criteria of children with CLP:**   1. Children diagnosed with cleft lip palate 2. aged of 7 and 12 years old 3. do not have any complex medical diagnosis 4. attend public primary school 5. understand, and speak fluent Malay language.   **Inclusion criteria of non-cleft comparison group:**   1. no history of language problems and other risk factors as reported by their parents 2. attended public school and used Malay as their main language   **Sources of recruitment:**   1. Cleft Lip and Palate Association Malaysia (CLAPAM 2. Hospital Canselor Tuanku Muhriz Universiti Kebangsaan Malaysia. |  |
|  |  | (*b*) *Cohort study*—For matched studies, give matching criteria and number of exposed and unexposed  *Case-control study*—For matched studies, give matching criteria and the number of controls per case | 7-8 | Control group:  - 25 age-matched children were recruited. These children had no history of language problems and other risk factors as reported by their parents and were randomly selected as a reference point (the non-cleft comparison group). All children attended public school and used Malay as their main lanaguage. |  |
| Variables | 7 | Clearly define all outcomes, exposures, predictors, potential confounders, and effect modifiers. Give diagnostic criteria, if applicable | 12 | Primary outcome measure was language performance (receptive language and expressive language). Independent variables or predictors were age, gender, hearing screening (pass/fail), types of hearing loss, maternal education, socio-economic status. |  |
| Data sources/ measurement | 8* | For each variable of interest, give sources of data and details of methods of assessment (measurement). Describe comparability of assessment methods if there is more than one group | 11 | The cut-off score of failing a subtest is based on the (Mean - 2SD) of each subtest from the non-cleft comparison group. |  |
| Bias | 9 | Describe any efforts to address potential sources of bias | 23 | Limitation section:  -Use of non-standardized Malay language test as there is no local language test or norms that designed for school-aged children.  -However, 25 children from the non-cleft comparison group were selected and the mean score of each test was used to compare with the CLP group to obtain valid language results and outcomes. |  |
| Study size | 10 | Explain how the study size was arrived at | 8-9 | Sample size was calculated using G*Power version 3.1.9.4 (Faul, Erdfelder, Lang, & Buchner, 2007). |  |

Continued on next page

| Quantitative variables | 11 | Explain how quantitative variables were handled in the analyses. If applicable, describe which groupings were chosen and why | 10-11 | Language outcomes and variables sub-headings. |
| --- | --- | --- | --- | --- |
| Statistical methods | 12 | (*a*) Describe all statistical methods, including those used to control for confounding | 11 | -Descriptive statistical analyses were used to describe the demographics section collected.  -Independent T-test was used to find the significant difference between the mean score of both groups.  -Multiple linear regression was used to determine the relationship between socio-demographic factors and language performance. |
|  |  | (*b*) Describe any methods used to examine subgroups and interactions | NA | NA |
|  |  | (*c*) Explain how missing data were addressed | NA | No missing data. |
|  |  | (*d*) *Cohort study*—If applicable, explain how loss to follow-up was addressed  *Case-control study*—If applicable, explain how matching of cases and controls was addressed  *Cross-sectional study*—If applicable, describe analytical methods taking account of sampling strategy | 11 | -Case control: Independent T-test was used to find the significant difference between the mean score of both groups.  -cross-sectional: Descriptive analysis |
|  |  | (*e*) Describe any sensitivity analyses | NA | NA |
| Results | | | | |
| Participants | 13* | (a) Report numbers of individuals at each stage of study—eg numbers potentially eligible, examined for eligibility, confirmed eligible, included in the study, completing follow-up, and analysed | 12 | Table 1 shows the demographic background of 52 school-aged children with CLP who participated in this study. |
|  |  | (b) Give reasons for non-participation at each stage | 12 | 1 participant withdrew from the study due to inability to complete the tests, leaving a total of 52 participants. |
|  |  | (c) Consider use of a flow diagram | NA | NA |
| Descriptive data | 14* | (a) Give characteristics of study participants (eg demographic, clinical, social) and information on exposures and potential confounders | 12-14 | The mean age of the total sample is 9.19 (SD = 1.61) years old. Refer manuscript. |
|  |  | (b) Indicate number of participants with missing data for each variable of interest | NA | NA |
|  |  | (c) *Cohort study*—Summarise follow-up time (eg, average and total amount) | NA | NA |
| Outcome data | 15* | Cohort study—Report numbers of outcome events or summary measures over time | NA | NA |
|  |  | *Case-control study—*Report numbers in each exposure category, or summary measures of exposure | 18 | Language Outcome  Table 5 and table 6 show the number of subjects being at risk or having language difficulty. |
|  |  | *Cross-sectional study—*Report numbers of outcome events or summary measures | *15-16* | -Language assessment  Table 2 shows the descriptive statistics of all language tasks administered, with the measures divided into receptive and expressive domains.  -Risk factors of language performance  Table 3 and table 4 show the unstandardised (B) and standardised (β) regression coefficients for each predictor in a regression model predicting total receptive and expressive language score. |
| Main results | 16 | (*a*) Give unadjusted estimates and, if applicable, confounder-adjusted estimates and their precision (eg, 95% confidence interval). Make clear which confounders were adjusted for and why they were included | NA | NA (No confounders in our study) |
|  |  | (*b*) Report category boundaries when continuous variables were categorized | NA | NA (We are reporting continuous data for language score, but not sure how to get the category boundaries) |
|  |  | (*c*) If relevant, consider translating estimates of relative risk into absolute risk for a meaningful time period | NA | NA |

Continued on next page

| Other analyses | 17 | Report other analyses done—eg analyses of subgroups and interactions, and sensitivity analyses | NA | NA |
| --- | --- | --- | --- | --- |
| Discussion | | | | |
| Key results | 18 | Summarise key results with reference to study objectives | 24 | **Objectives 1 & 2:**  The results indicated that children with CLP in this study showed differences in language performance. Specifically, on the reading comprehension (p = 0.023) and narrative (p = 0.024) tasks, while yielding similar performances on the remaining grammatical understanding, listening comprehension and sentence repetition tasks.  **Objective 3:**  The age significantly influenced total receptive language score (β = 0.421, p = 0.003) and total expressive language score (β = 0.477, p = 0.000) in school-aged children with CLP. |
| Limitations | 19 | Discuss limitations of the study, taking into account sources of potential bias or imprecision. Discuss both direction and magnitude of any potential bias | 28-30 | -The use of non-standardized test tools in Malay for assessing language skills in this study and there are no norms for the tests that can be compared to the school-aged children with CLP.  -The non-cleft comparison group participants were chosen randomly and reported to be typically developing language skills with no clinical evaluation or assessment of their language skills.  -Language tests were carried out through physical and online sessions based on the participants’ availability. |
| Interpretation | 20 | Give a cautious overall interpretation of results considering objectives, limitations, multiplicity of analyses, results from similar studies, and other relevant evidence | 30 | Conclusion |
| Generalisability | 21 | Discuss the generalisability (external validity) of the study results | 30 | Our study highlighted the need to raise awareness among healthcare professionals especially speech language therapists to include the language assessment and intervention in management of school-aged children with CLP. |
| Other information | |  | | |
| Funding | 22 | Give the source of funding and the role of the funders for the present study and, if applicable, for the original study on which the present article is based | 31 | This research was funded by a grant from the Ministry of Higher Education- Fundamental Research Grant Scheme (FRGS/1/ 2021/SS0/UKM/02/2). |

*Give information separately for cases and controls in case-control studies and, if applicable, for exposed and unexposed groups in cohort and cross-sectional studies.

**Note:** An Explanation and Elaboration article discusses each checklist item and gives methodological background and published examples of transparent reporting. The STROBE checklist is best used in conjunction with this article (freely available on the Web sites of PLoS Medicine at http://www.plosmedicine.org/, Annals of Internal Medicine at http://www.annals.org/, and Epidemiology at http://www.epidem.com/). Information on the STROBE Initiative is available at www.strobe-statement.org.
