# Supplementary figures and images for: Language abilities and associated risk factors of school-aged children with cleft lip and palate
Source: PLoS One. 2024 Apr 22;19(4):e0299095. doi: 10.1371/journal.pone.0299095 (PMC11034652; doi:10.1371/journal.pone.0299095)

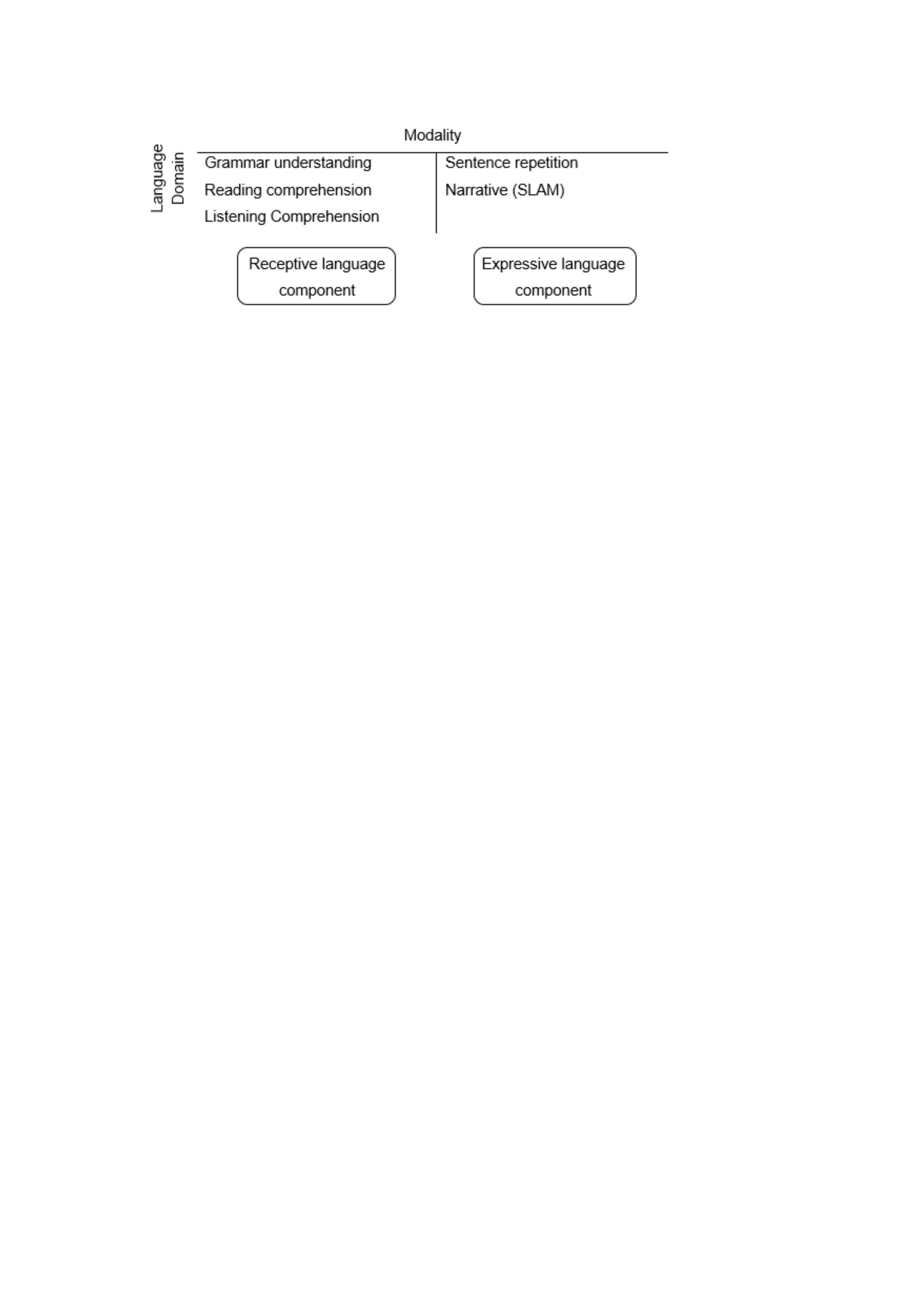

Supplement: S1 Fig — (TIF) [file pone.0299095.s004.tif]
